# Prevention of excessive exercise‐induced adverse effects in rats with *Bacillus subtilis* BSB3

**DOI:** 10.1111/jam.14544

**Published:** 2019-12-25

**Authors:** H.A.G. Ducray, L. Globa, O. Pustovyy, M.D. Roberts, M. Rudisill, V. Vodyanoy, I. Sorokulova

**Affiliations:** ^1^ Department of Anatomy, Physiology and Pharmacology Auburn University Auburn AL USA; ^2^ School of Kinesiology Auburn University Auburn AL USA

**Keywords:** *Bacillus subtilis*, gut microbiota, I‐FABP, LPS, side effects of excessive exercise, tight junction proteins

## Abstract

**Aims:**

To characterize efficacy of the *Bacillus subtilis* BSB3 (BSB3) strain in the prevention of excessive exercise‐induced side effects and in maintaining stability of the gut microbiota.

**Methods and Results:**

Rats were pretreated by oral gavage with *B. subtilis* BSB3 (BSB3) or with phosphate‐buffered saline (PBS) twice a day for 2 days, and were either exposed forced treadmill running or remained sedentary. Histological analysis of intestine, immunofluorescence staining of tight junction (TJ) proteins, serum lipopolysaccharide and intestinal fatty acid‐binding protein assay, culture‐based analysis and pyrosequencing for the gut microbiota were performed for each rat. Forced running resulted in a substantial decrease in intestinal villi height and total mucosa thickness, the depletion of Paneth cells, an inhibition of TJ proteins expression. Short‐term treatment of rats with BSB3 before running prevented these adverse effects. Culture‐based analysis of the gut microbiota revealed significant elevation of pathogenic microorganisms only in treadmill‐exercised rats pretreated with PBS. High‐throughput 16S rRNA gene sequencing also revealed an increase in pathobionts in this group. Preventive treatment of animals with BSB3 resulted in predominance of beneficial bacteria.

**Conclusions:**

BSB3 prevents excessive exercise‐associated complications by beneficial modulation of the gut microbiota.

**Significance and Impact of the Study:**

Our study shows a new application of beneficial bacteria for prevention the adverse effects of excessive exercise.

## Introduction

Physical activity induces distinct effects on health depending on intensity and duration. Regular moderate exercise reduces the incidence of inflammatory diseases, has protective effect against cancer, gastrointestinal disorders and obesity (Martin 2011; Kruk and Czerniak 2013; Allen *et al.* 2018). Physical activity is associated with a marked decrease in all‐cause mortality, cardiovascular mortality, cardiovascular disease (Kraus *et al.* 2019) and with a delay in onset of dementia in elderly persons (Larson *et al.* 2006). However, recent data suggest high level of physical activity results in increased risk of early mortality (Coenen *et al.* 2018). Excessive exercise has also been associated with a significant immunodepression, causing lower resistance to pathogens and an increase in the risk of infections and illnesses (Castell *et al.* 2019).

Intestinal complications are the most common among endurance athletes (Pires *et al.* 2017). To this end, 20–60% of athletes report various abdominal symptoms, such as bloating, nausea, vomiting, stomach pain, flatulence, diarrhoea, and constipation following a strenuous exercise bout (de Oliveira and Burini 2009; Pane *et al.* 2018). It has been suggested that endurance exercise reduces gastrointestinal blood flow, increases gut permeability and translocation of the gut microbiota and its metabolites into circulation (de Oliveira and Burini 2009). Recent scientific data indicate the significant role of the gut microbiota in host physiology, particularly in the maintenance of metabolism, immune homeostasis and mental health (Sommer and Backhed 2013; Naseribafrouei *et al.* 2014; Rieder *et al.* 2017; Cani 2018; Dicks *et al.* 2018). Stability of the gut microbiota is essential for the healthy status of the host, as disease conditions are accompanied with dysbiosis (Pham and Law 2014; Sommer *et al.* 2017). Significant changes in the gut microbiota with prevalence of conditionally pathogenic bacteria, similar to those induced by a high fat diet, have been observed after forced exercise (Kang *et al.* 2014; Allen *et al.* 2015). Balanced gut microbiota is responsible for normal gut morphology and function, whereas dysbiosis results in significant increase in gut permeability and inflammation (Dicks *et al.* 2018). Thus, maintaining the balanced gut microbiota can prevent various adverse effects associated with dysbiosis.

Probiotics have been suggested as a valuable approach for normalization of the gut microbiota with the intent of preventing and treating various pathological disorders (O'toole and Cooney 2008; Aureli *et al.* 2011). Special attention is paid for probiotic supplementation in sport medicine for prevention health issues, affecting sport performance, especially in endurance athletes. In clinical trials, some probiotics were effective in reducing incidence of upper respiratory symptoms and gastrointestinal problems in athletes, although other studies did not show efficacy (Leite *et al.* 2019). These clinical trials were focused on analysis of specific symptoms and immunological data, but no gut microbiota studies have been performed. Our previous results showed high efficacy of *Bacillus subtilis* BSB3 (BSB3) strain in prevention of heat stress‐related adverse events in rats (Moore *et al.* 2014; Sorokulova *et al.* 2016). However, it is currently unknown if BSB3 can affect biomarkers associated with gastrointestinal health following a rigorous bout of exercise. Therefore, the main aim of recent study was to characterize proficiency of this strain in mitigation side effects of excessive exercise and in maintaining stability of the gut microbiota in rats.

## Materials and methods

### Ethics statement

All animal procedures were approved by the Auburn University Institutional Animal Care and Use Committee (protocol number 2016‐2940 ‘Efficacy of a preexercise probiotic intervention on mitigating the adverse effects resulting from metabolic‐derived heat stress’, 09/08/2016). The study was performed in accordance with the Guide for the Care and Use of Laboratory Animals of the National Institutes of Health.

### Animals

Adult male Sprague–Dawley rats (Harlan Laboratories, Indianapolis, IN) weighing 250–300 g were used in this study. Sprague–Dawley rats were used in our previous studies (Moore *et al.* 2014; Ducray *et al.* 2019) and in the studies of other authors (Lambert *et al.* 2002; Gong *et al.* 2012). Animals were housed two per cage under specific pathogen‐free conditions and were acclimatized for 2 days prior to experimentation at a temperature of 20 ± 1°C and standard lighting (12‐h day/12‐h night) with free access to water and standard food (2018 Teklad Global 18% Protein Rodent Diet; Harlan).

### Bacterial culture

*Bacillus subtilis* BSB3 was cultivated on plates with Difco sporulation medium (Difco Nutrient Broth; Becton, Dickinson and Company, Sparks, MD) at 37°C for 5 days. Bacteria were harvested by flooding the surface of the plates with sterile phosphate‐buffered saline (PBS) followed by scraping with a sterile cell spreader. The bacterial suspension was diluted in PBS to achieve 1 × 10^8^ colony‐forming unit (CFU) per ml.

### Antibodies

Primary rabbit polyclonal antibodies against zonula occludens (ZO‐1) (#61‐7300), occludin (#40‐4700), claudin (#37‐4900), Alexa Fluor 555 goat anti‐rabbit (#A32727) and Alexa Fluor 488 goat anti‐mouse (#A32723) secondary antibodies were acquired from ThermoFisher Scientific (Waltham, MA). Rabbit Anti‐Junctional Adhesion Molecule 1 (JAM‐A antibody, #ab125886) were from Abcam (Cambridge, MA).

### Experimental design

Animals (*n* = 24) were treated twice a day with 6‐h dose intervals by oral gavage either with 1 ml of *B. subtilis* BSB3 suspension (*n* = 12) or 1 ml of PBS (*n* = 12) for 2 days. On day 3, rats in each group were further subdivided: BEx—pretreated with BSB3, undergoing forced running; BCont—pretreated with BSB3 remaining sedentary; PEx—pretreated with PBS, undergoing forced running; PCont—pretreated with PBS remaining sedentary. The forced running protocol was performed according to Przyborowski *et al. *(2017) with modifications. Briefly, animals started running on a treadmill (Exer‐3/6 Treadmill; Columbus Instruments, Columbus, OH) at 5 m min^−1^ followed by gradual increases in speed of 2 m min^−1^ until exhaustion. Rectal temperature was measured for each rat before and immediately after forced running using an electronic digital thermometer (Ducray *et al.* 2016). Four hours after the forced running experiments, rats were anaesthetized with isoflurane (2–4%) and killed by rapid decapitation. Trunk blood was collected from each rat into sterile apirogenic tubes to obtain serum. Sections of small intestine from each rat were taken for morphological analysis. Colon faecal matter was immediately placed in anaerobic broth for culture‐based microbiological analysis. For 16S rRNA sequencing of the gut microbiota faecal samples were placed into sterile tubes and stored at −80°C until the experiment.

### Blood serum preparation

Blood collected in sterile tubes was allowed to clot for 30 min at room temperature. Tubes were centrifuged at 20°C, 7000 ***g*** for 10 min. Serum was collected and stored in 50 µl aliquots at −20°C until assay.

### Histological analysis

#### Sample preparation

Small intestinal samples (0·5–2 cm in length) were completely immersed in fixative, Bouin's solution (Electron Microscopy Sciences, Hatfield, PA), immediately after harvesting. After 48 h of fixation at room temperature, the excess fixative was washed out in 70% ethanol (ETOH). Washed samples were placed into tissue embedding cassettes (VWR, Radnor, PA) and kept in 70% ETOH until processing in the Automated tissue processor (Tissue‐Tek VIP; Miles/Sakura, Torrance, CA). After processing, samples were embedded in paraffin blocks using embedding centre (Tissue‐Tek TEC; Sakura). Embedded tissues were sectioned at 6 mm using a microtome (Reichert‐Jung 2040 Autocut; Leica Biosystems Nussloch GmbH, Nussloch, Germany) and then mounted on slides until staining.

#### Sample staining

Histological sections were deparaffinized, dehydrated and stained with haematoxylin and eosin according to the standard protocol (Stevens 1990). After staining, sections were mounted using Eukitt Mounting Medium (Electron Microscopy Sciences, Hatfield, PA).

#### Measurements

Intestinal villi height and total mucosal thickness for each sample were measured as previously described (Ducray *et al.* 2016). Twenty measurements of each parameter in each sample were taken and expressed in micrometres. A total of eight sections were counted per rat for each rat in the treatment group. An average of these measurements was expressed as a mean villi height and mean total mucosal thickness for one treatment group.

### Goblet cells count

Four sections of the small intestine from each rat were stained with Alcian Blue using a standard procedure, as previously described (Ducray *et al.* 2019). Enumeration of goblet cells was according the protocol proposed by Trevizan *et al. *(2016). Six images from each section were taken with a digital camera (Olympus BX50) coupled to an optical microscope with a 20x objective. The number of goblet cells present in a 0·96 mm^2^ in the mucosa of each animal were quantified using ImagePro 10 software (Media Cybernetics, Rockville, MD).

### Paneth cells quantification

Paneth cells were visualized by phloxine‐tartrazine staining (Di Sabatino *et al.* 2008). Briefly, four sections of each rat were treated with Gill’s haematoxylin, phloxine B‐calcium carbonate, saturated solution of tartrazine. Quantification of Paneth cells was performed for each sample using a high‐resolution microscope system (Vainrub *et al.* 2006). Only crypts cut along the length of the crypt lumen were analysed.

### Blood serum analysis

Lipopolysaccharide (LPS) serum concentration was analysed by the Pierce LAL Chromogenic Endotoxin Quantitation Kit (Thermo Scientific, Rockford, IL) using the limulus amebocyte lysate assay according to the manufacturer's recommendations. The sensitivity of the assay was 0·1 EU ml^−1^ (0·01 ng endotoxin per millilitres).

Concentration of intestinal fatty acid‐binding protein (I‐FABP) was determined by Rat I‐FABP/FABP2 ELISA Kit (LifeSpan BioSciences, Seattle, WA) with sensitivity <0·094 ng ml^−1^ according to the manufacturer's instruction.

### Immunofluorescence staining of junctional proteins

Histological sections were deparaffinized in Hemo‐Di x 2 changes for 5 min, hydrated with 100, 95 and 70% ETOH and distilled water. Sections were blocked for 1 h in Odyssey blocking buffer (LiCor, Lincoln, CA) and incubated for 2 h at 4°C with primary antibodies against claudin, occludin, ZO‐1 and JAM‐A proteins. Sections were washed with PBS and incubated with secondary antibodies (1 : 100 dilution) for 1 h, then washed with PBS/0·01% Tween. Sections were mounted with VectaShield Mounting Media (Vector Laboratories, Burlingame, CA). Fluorescence images from each section were obtained with a digital camera (Olympus BX50) coupled to an optical microscope. The images were analysed with Image‐Pro10 (Media Cybernetics). Three fields per each of three sections from each rat were analysed.

### Gut microbiota analysis

#### Culture‐based study

Faecal matter removed from the colon of each rat using sterile technique, was placed in sterile preweighted tubes with anaerobic broth, weighted and vortexed until homogenous. Serial tenfold dilutions from 10^−1^ to 10^−7^ were prepared and from the appropriate dilution, 0·1 ml aliquots were plated in four replicates on each of media: Anaerobic Basal Agar (Alfa Aesar, Ward Hill, MA) for total anaerobic bacteria; Brain Heart agar (Hardy Diagnostics, Santa Maria, CA) for total aerobic bacteria; Trypticase Soy Agar with Sheep Blood (Fisher Scientific, Hampton, NH) for haemolytic bacteria; *Bifidobacterium* Selective Agar (HiMedia Laboratories, Nashik, MH) for *Bifibacterium* sp.; Difco Lactobacilli MRS agar (Becton Dickinson) for *Lactobacillus* sp.; Brucella Agar w/Hemin and vitamin K1 (HiMedia Laboratories) for *Bacteroides* sp.: Reinforced Clostridial Medium (Hardy Diagnostics, Santa Maria, CA) for *Clostridia* sp.; Sabouraud Dextrose HiVeg Agar (HiMedia Laboratories) for yeasts. For isolation of anaerobic bacteria, plates were placed in an anaerobic chamber in a microaerophilic environment generated by a GasPak EZ Anaerobe Container System (Becton Dickinson and Co). All plates were incubated at 37°C and checked after 24 h for aerobic bacteria counting and after 48 h—for anaerobic bacteria. The number of CFU per gram of faecal matter was determined. Bacterial cultures and yeasts were identified by morphology of colonies, microscopical analysis of cells’ morphology, Gram staining, formation of spores, aerobic and anaerobic growth, as it was recommended elsewhere (Benno and Mitsuoka 1992; Sudo *et al.* 2004).

#### Pyrosequencing

Faecal samples were submitted to MR DNA (Shallowater, TX) for DNA isolation and sequencing. Analysis of the 16S rDNA was performed according to a previously published procedure (Dowd *et al.* 2008). Total genomic DNA was extracted from faecal samples using a QIAamp stool DNA (Qiagen, Germantown, MD) according the manufacture’s instruction. DNA samples were quantified using a Nanodrop spectrophotometer (Nyxor Biotech, Paris, France). The 16S rRNA gene V4 variable region PCR primers 515F GTGCCAGCMGCCGCGGTAA and 806R GGACTACHVGGGTWTCTAAT were utilized to evaluate the microbial ecology of each sample on the HiSeq 2500 with methods via the bTEFAP DNA analysis service. Each sample underwent a single‐step 30 cycle PCR using HotStarTaq Plus Master Mix Kit (Qiagen, Valencia, CA) under the following conditions: 94°C for 3 min, followed by 28 cycles of 94°C for 30 s; 53°C for 40 s and 72°C for 1 min; after which a final elongation step at 72°C for 5 min was performed. Following PCR, each stochastic replicate was combined and all amplicon products from different samples were mixed in equal concentrations and purified using Agencourt Ampure beads (Agencourt Bioscience Corporation, MA). Samples were sequenced utilizing the Illumina HiSeq chemistry following manufacturer’s protocols. The sequence data were processed using a proprietary analysis pipeline (MR DNA, Shallowater). Sequences smaller than 200 base pairs, contained ambiguous base calls, or contained homopolymer runs exceeding six base pairs were removed. Operational taxonomic units (OTU) were defined after removal of singleton sequences, clustering at 3% divergence (97% similarity). OTU were taxonomically classified using BLASTn against a curated National Center for Biotechnology Information database.

### Statistics

All results were presented as a mean and standard deviation, unless otherwise indicated. Differences between groups were analysed by the one‐way anova, followed by the Fisher *post hocs*. Statistical analysis of sequence results was performed using a variety of computer packages including XLstat, NCSS 2007, ‘R’ and NCSS 2010. Significance reported for any analysis is defined as *P* < 0·05. Statistical calculations and graph plotting were carried out using Microcal Origin ver. 9.0 (Northampton, MA) and 2010 Microsoft Excel.

## Results

### Body temperature

Forced running resulted in significant elevation of rats’ body temperatures. Specifically, the mean body temperature was 36·7 ± 0·6°C prior to exercise and 39·3 ± 0·3°C immediately after exercise (*P* < 0·05). Body temperatures of the control (sedentary) rats were not altered (37·3 ± 0·4°C *vs* 37·6 ± 0·6°C).

### Intestinal morphometry

**Figure 1:**
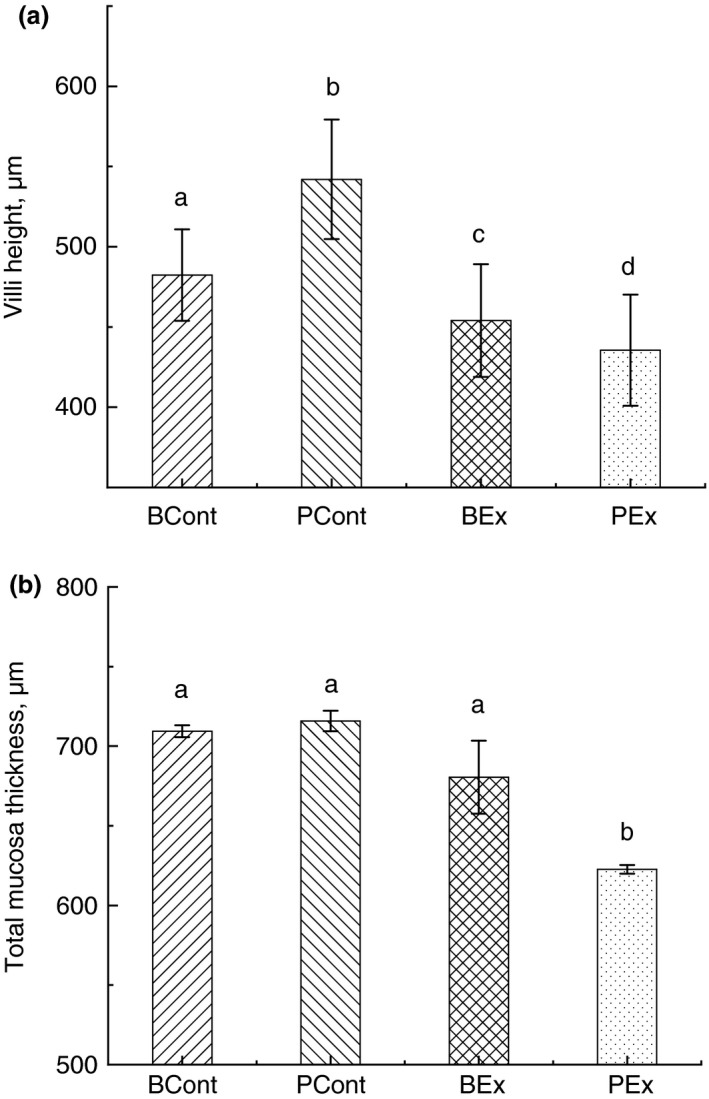
Intestinal villi height (a) and total mucosa thickness (b) in rats from different experimental groups. Rats were pretreated by oral gavage with *Bacillus subtilis* BSB3 (BSB3) or with PBS and were either exposed forced treadmill running or remained sedentary (*n* = 6 per condition). BEx, pretreated with BSB3, undergoing forced running; BCont, pre‐treated with BSB3 remaining sedentary; PEx, pretreated with PBS, undergoing forced running; PCont, pretreated with PBS remaining sedentary. Data with different superscript letters signify groups are significantly different (*P* < 0·05).

Microscopic analysis of the small intestine revealed a significant decrease in villus height and total mucosa thickness in rats pretreated with PBS and exposed to forced running (PEx group) in comparison with rats from control PCont group (Fig. 1a,b). Villi height in animals from PEx and PCont groups were 435·57 ± 34·64 µm and 531·21 ± 26·11 µm (*P* < 0·05), and total mucosa thickness in these groups were 622·67 ± 15·27 µm and 715·83 ± 35·72 µm (*P* < 0·05) accordingly. Pretreatment of rats with BSB3 prior to forced running (BEx group) also resulted in significant reductions of the villi height in comparison with control groups (PCont and BCont), but values were significantly higher in comparison with animals from PEx group (531·21 ± 26·11 µm *vs* 435·57 ± 34·64 µm; *P* < 0·05). No significant reduction in the total mucosa thickness was observed in the BEx group in comparison with control groups.

### Paneth cells

**Figure 2:**
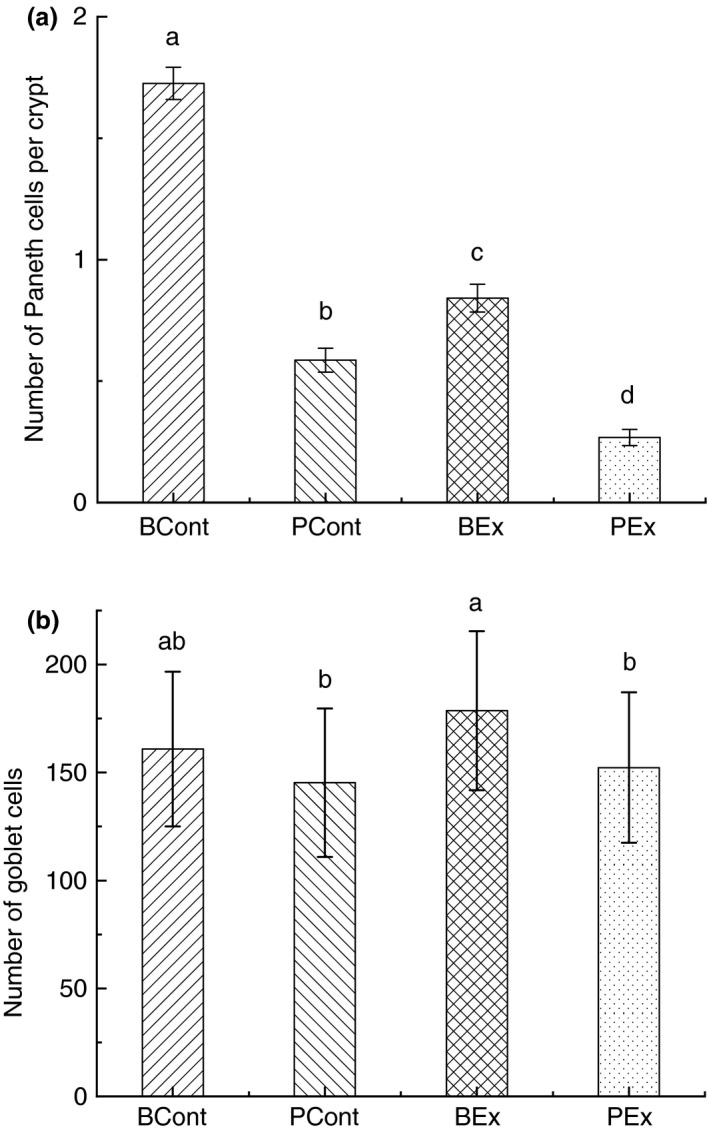
Paneth (a) and goblet (b) cells in intestine of rats from different experimental groups. Rats were pretreated by oral gavage with *Bacillus subtilis* BSB3 (BSB3) or with PBS and were either exposed forced running or remained sedentary (*n* = 6 rats per condition). BEx, pretreated with BSB3, undergoing forced running; BCont, pretreated with BSB3 remaining sedentary; PEx, pretreated with PBS, undergoing forced running; PCont, pretreated with PBS remaining sedentary. Data with different superscript letters signify groups are significantly different (*P* < 0·05).

Forced running resulted in significant reductions of Paneth cells number in rats pretreated with PBS (PEx group) in comparison with control PCont group (0·27 ± 0·03 and 0·67 ± 0·06 respectively; *P* < 0·05) (Fig. 2a). Rats administered BSB3 before forced running (BEx group) demonstrated significantly higher counts of Paneth cells in comparison with PEx group (0·93 ± 0·06 *vs* 0·27 ± 0·03, *P* < 0·05). Pretreatment of control rats with BSB3 (BCont) resulted in significantly higher numbers of Paneth cells when compared with rats from the PCont group (1·78 ± 0·07 and 0·67 ± 0·06 accordingly, *P* < 0·05).

### Goblet cells

Pretreatment of rats with BSB3 prior to forced running (BEx) resulted in significant increase in goblet cells in comparison with rats pretreated with PBS (PEx and PCont groups). Specifically, the number of goblet cells in BEx, PEx and PCont animals was 178·63 ± 36·82, 152·29 ± 34·79 and 145·29 ± 34·43 respectively (Fig. 2b).

### Expression of TJ proteins

**Figure 3:**
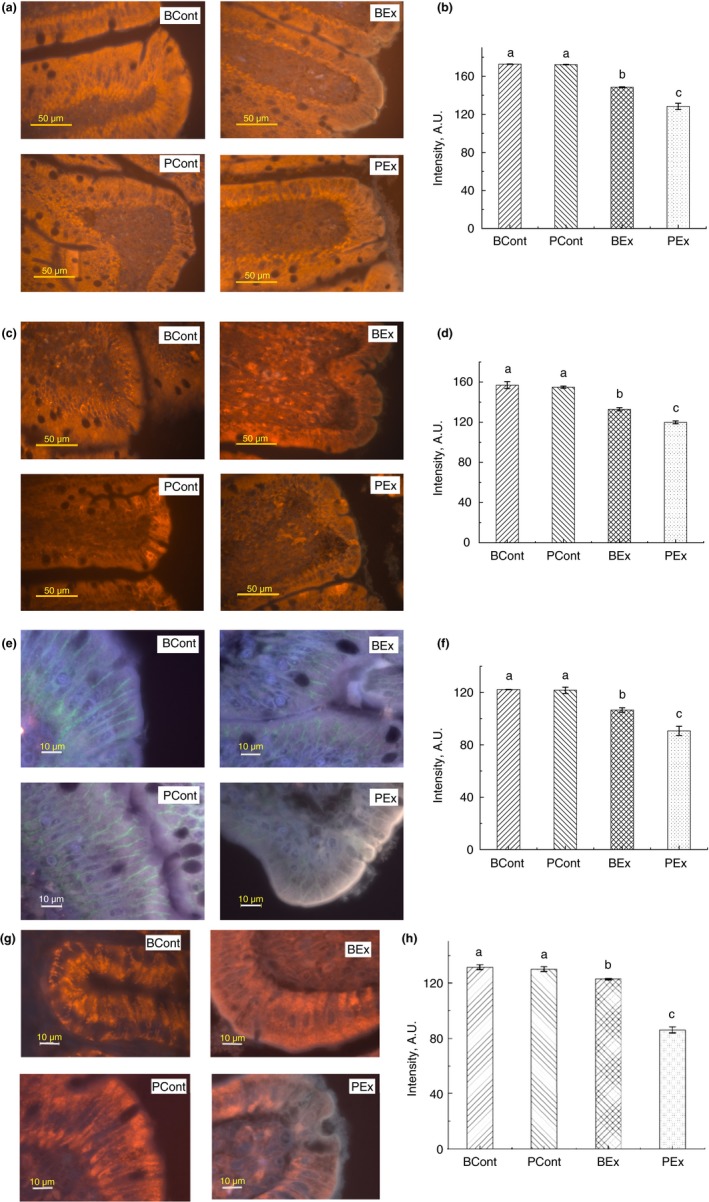
Immunofluorescence staining of TJ proteins in the intestine of rats from different experimental groups. (a) Images of occludin immunostaining, Bar 50 µm; (b) Analysis of occludin expression with immunostaining; (c) Images of ZO‐1 immunostaining, bar 50 µm; (d) Analysis of ZO‐1 expression with immunostaining; (e) Images of claudin immunostaining, bar 10 µm; (f) Analysis of claudin expression with immunostaining; (g) Images of JAM‐A immunostaining, bar 10 µm; (h) Analysis of JAM‐A expression with immunostaining. A.U., arbitrary units. Rats were pretreated by oral gavage with *B. subtilis* BSB3 (BSB3) or with PBS and were either exposed forced running or remained sedentary (*n* = 6 rats per condition). BEx, pretreated with BSB3, undergoing forced running; BCont, pretreated with BSB3 remaining sedentary; PEx, pretreated with PBS, undergoing forced running; PCont, pretreated with PBS remaining sedentary. Data with different superscript letters signify groups are significantly different (*P* < 0·05). [Colour figure can be viewed at https://www.wileyonlinelibrary.com]

Levels of all assayed tight junction (TJ) proteins were significantly lower in PEx rats in comparison with all other groups (Fig. 3). Pretreatment with BSB3 before forced running resulted in a considerable increase in the expression of TJ proteins compared to the PEx group, though these values were lower than control groups.

### Serum LPS concentration

**Figure 4:**
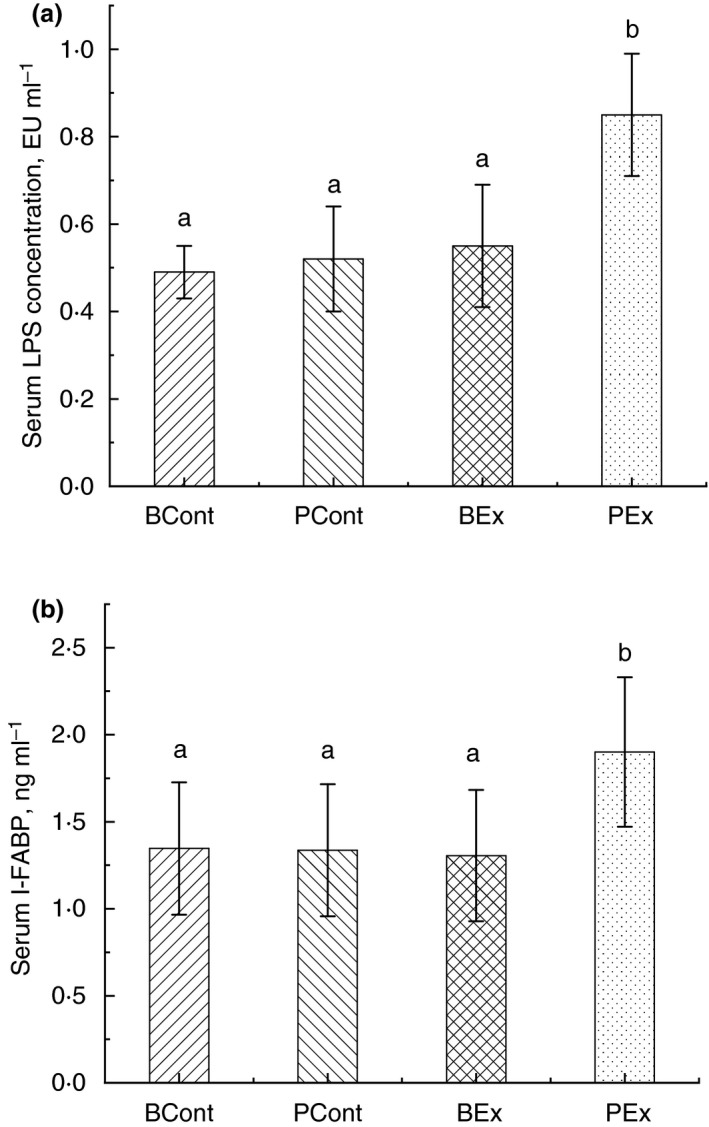
Serum LPS (a) and I‐FABP (b) concentration in rats from different experimental groups. Rats were pretreated by oral gavage with *Bacillus subtilis* BSB3 (BSB3) or with PBS and were either exposed forced running or remained sedentary (*n* = 6 rats per condition). BEx, pretreated with BSB3, undergoing forced running; BCont, pretreated with BSB3 remaining sedentary; PEx, pretreated with PBS, undergoing forced running; PCont, pretreated with PBS remaining sedentary. Data with different superscript letters signify groups are significantly different (*P* < 0·05).

Significant elevation of serum LPS level was observed only in treadmill‐exercised rats pretreated with PBS (PEx group). Treatment with BSB3 prevented rise of LPS in BEx animals. Specifically, serum LPS concentrations in PEx and BEx rats were 0·85 ± 0·14 and 0·55 ± 0·12 EU ml^−1^ accordingly (*P* < 0·05). (Fig. 4a).

### I‐FABP

Considerably elevated level of serum I‐FABP (1·9 ± 0·4 ng ml^−1^) detected only in rats, received PBS before forced running. Concentration of I‐FABP in animals from BEx group was the same as in control rats (1·3 ± 0·3 ng ml^−1^) (Fig. 4b).

### Gut microbiota analysis

#### Culture‐based analysis

**Figure 5:**
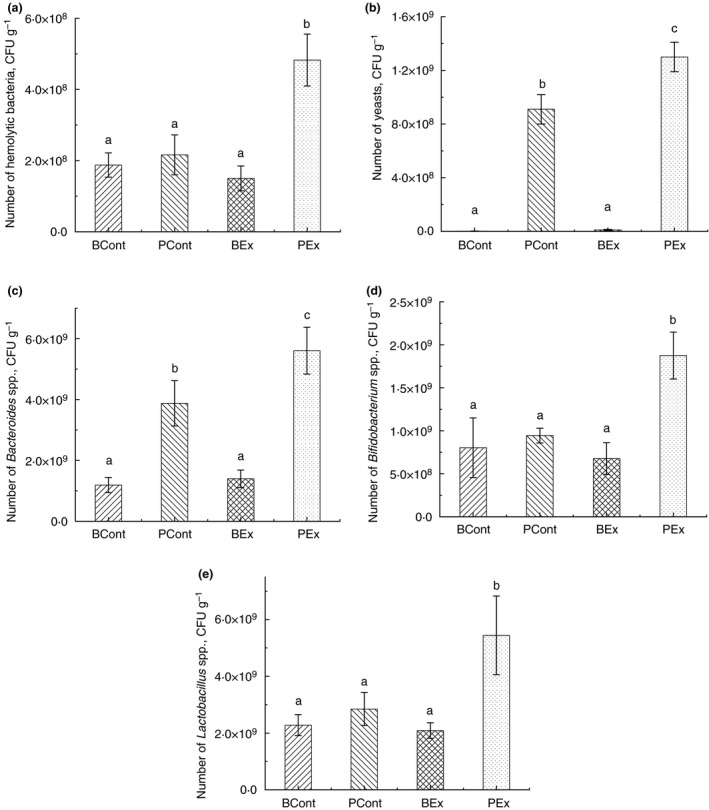
Results of the gut microbiota analysis by a culture‐based method: (a) Number of hemolytic bacteria; (b) Number of yeasts; (c) Number of *Bacteroides* spp.; (d) Number of *Bifidobacterium* spp.; (e) Number of *Lactobacillus* spp. Rats were pretreated by oral gavage with *Bacillus subtilis* BSB3 (BSB3) or with PBS and were either exposed forced running or remained sedentary (*n* = 6 rats per condition). BEx, pretreated with BSB3, undergoing forced running; BCont, pretreated with BSB3 remaining sedentary; PEx, pretreated with PBS, undergoing forced running; PCont, pretreated with PBS remaining sedentary. Data with different superscript letters signify groups are significantly different (*P* < 0·05).

Culture‐based analysis of the gut microbiota showed a significant elevation of haemolytic bacteria, yeasts and *Bacteroides* sp. in rats from the PEx group compared with other groups (Fig. 5a). Yeasts and *Bacteroides* sp. counts was higher in control rats, pretreated with PBS (PCont group) compared to rats that obtained BSB3 (BCont and BEx groups) (Fig. 5b,c). Considerably higher numbers of *Lactobacillus* sp. and *Bifidobacterium* sp. were revealed in rats from the PEx group (Fig. 5d,e). Treatment with BSB3 did not affect these bacteria counts in comparison with control PCont rats.

#### Sequencing

After stringent quality sequence curation, a total of 2 251 607 sequences were parsed and 2 074 232 were then clustered. 2 073 720 sequences identified within the Bacteria domain were utilized for final microbiota analyses. The average reads per sample was 129 607.

**Figure 6:**
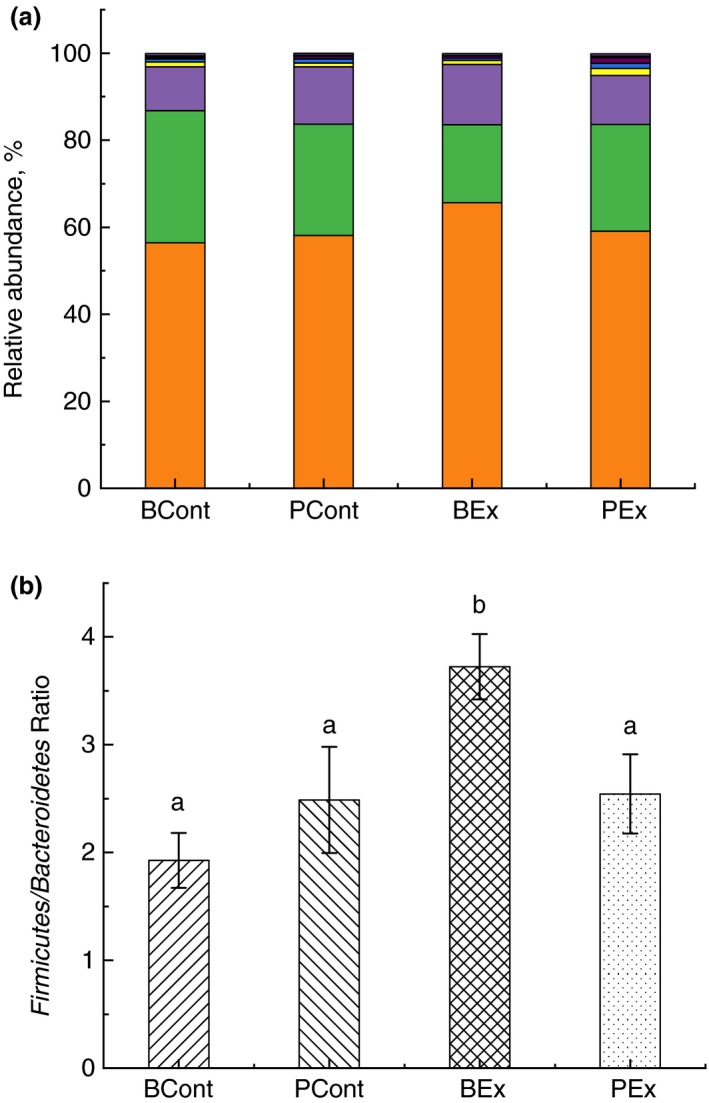
Analysis of the gut microbiota composition of rats at the phylum level. (a) Relative abundance of phyla. All phyla present in abundance of <0·1% are presented as other (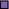 Others; 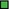 *Cyanobacteria*; 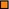 TM7; 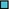 *Elusimicrobio*; 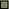 *Deferribacteres*; 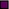 *Verrucomicrobia*; 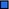 *Proteobacteria*; 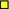 *Tenericutes*; 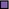 *Actinobacteria*; 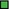 *Bacteroidetes*; 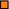 *Firmicutes*); (b) *Firmicutes* to *Bacteroidetes* ratio. Rats were pretreated by oral gavage with *Bacillus subtilis* BSB3 (BSB3) or with PBS and were either exposed forced running or remained sedentary (*n* = 6 rats per condition). BEx, pretreated with BSB3, undergoing forced running; BCont, pretreated with BSB3 remaining sedentary; PEx, pretreated with PBS, undergoing forced running; PCont, pretreated with PBS remaining sedentary. Data with different superscript letters signify groups are significantly different (*P* < 0·05). [Colour figure can be viewed at https://www.wileyonlinelibrary.com]

Ten bacterial phyla were detected in rats from all groups (Fig. 6a) with three dominant phyla: *Firm*icutes, *Bacteroidetes* and *Actinobacteria.* No significant difference between phyla was found, but the *Firmicutes* to *Bacteroidetes* ratio in the BEx group showed marked distinction from all other groups (Fig. 6b). More notable changes were observed at the genus level (Table 1). Comparison of the gut microbiota in PEx rats with the PCont group revealed significant depletion of *Candidatus arthromitus*, elevation of *Veillonella* and appearance of *Facklamia.* Pretreatment of rats with BSB3 before forced running (BEx group) resulted in considerable increase in *Allobaculum*, *Faecalibacterium, Olsenella* and decrease in *C. arthromitus* and *Flavonifractor* in comparison with PCont rats. Substantial elevation of *Olsenella* was found also in control rats, pretreated with BSB3 (BCont group). Microbial composition of the gut microbiota in this group was different from the PCont group by significant decrease in *Parasporobacterium*.

**Table 1:** Changes of the genera in the gut microbiota of rats from different experimental groups

Genus	PCont		PEx		PEx *vs* PCont changes, %	*P* value
	Mean	SEM	Mean	SEM		
*Candidatus arthromitus*	0·0575	0·0119	0·0061	0·0038	−89·4505	0·0147
*Enterorhabdus*	0·0083	0·0005	0·0164	0·0020	97·2746	0·0176
*Facklamia*			0·0016	0·0008	PEx*	
*Veillonella*			0·0022	0·0015	PEx*	

Genus	PCont		BEx		BEx *vs* PCont changes, %	P value
	Mean	SEM	Mean	SEM		
*Allobaculum*	0·0289	0·0092	0·4302	0·0326	1390·7571	0·0002
*Candidatus arthromitus*	0·0575	0·0119	0·0181	0·0075	−68·5834	0·0487
*Faecalibacterium*	0·0143	0·0056	0·0331	0·0038	131·3225	0·0411
*Flavonifractor*	0·0469	0·0051	0·0224	0·0072	−52·3085	0·0499
*Olsenella*	0·0104	0·0024	0·1998	0·0501	1830·1272	0·0241

Genus	PCont		BCont		BCont *vs* PCont changes, %	*P* value
	Mean	SEM	Mean	SEM		
*Olsenella*	0·0104	0·0024	0·1729	0·0281	1569·6716	0·0045
*Parasporobacterium*	0·4358	0·0763	0·1613	0·0326	−62·9872	0·0297

Rats were pretreated by oral gavage with *B. subtilis* BSB3 (BSB3) or with PBS and were either exposed forced running or remained sedentary (*n* = 6 rats per condition).

BEx, pretreated with BSB3, undergoing forced running; BCont, pretreated with BSB3 remaining sedentary; PEx, pretreated with PBS, undergoing forced running; PCont, pretreated with PBS remaining sedentary.

* Genus was found only in this group.

## Discussion

The results of this study demonstrated that pretreatment of rats with *B. subtilis* BSB3 bacteria prevented adverse effects, associated with an exhaustive treadmill exercise bout through beneficial modulation of the gut microbiota.

Our data showed that core body temperature in rats, exposed to exhaustive treadmill exercise exceeded 39°C, which was significantly higher than in sedentary controls. These data indicate that heat stress occurred during exercise without an elevation in environmental temperatures, and these elevations were similar to values obtained in our previous study, which involved increasing core body temperatures via environmental heat stress without exercise (Moore *et al.* 2014).

We found the changes in gut morphology in rats, pretreated with PBS before forced running (PEx group). Thus, villi height and total mucosa thickness in this group significantly decreased in comparison with control sedentary animals. BSB3 treatment of rats before exhaustive treadmill exercise (BEx group) prevented substantial gut injury: mucosa thickness did not change compared to control BCont and PCont groups; villi height significantly increased in comparison with PEx group. These results agree with our previous findings, in that elevated core body temperature is accompanied by considerable decrease in villi height and BSB3 administration can prevent this damage to the gut of rats (Moore *et al.* 2014).

Further analysis revealed reduction in Paneth cells in PEx rats, but BSB3 administration prevented the exercise‐induced depletion of these cells. Paneth cells are specialized secretory cells which produce antimicrobial peptides and other compounds, that contribute to intestinal homeostasis and rejuvenation of the intestinal epithelium (Bevins and Salzman 2011; Clevers and Bevins 2013). A reduction in Paneth cells may result in development of epithelial barrier defects, multiorgan dysfunction and systemic inflammation (Estienne *et al.* 2010; Park *et al.* 2012). Heat stress is one of the factors responsible for a decrease in Paneth cell number, as we have demonstrated this in a previous study (Ducray *et al.* 2019). We did not observe a decrease in the number of goblet cells in PEx rats compared to sedentary controls. This finding is consistent with prior studies which have demonstrated no changes in goblet cell number after physical activity (Remedio *et al.* 2012) or high‐intensity training (Gong *et al.* 2012).

Notwithstanding, goblet cells significantly increased in rats administered BSB3 prior to treadmill running. Goblet cells are a major producer of mucin, which maintains the integrity of protective mucus barrier, and is a first line of innate defence (Kim and Ho 2010). We suggest that BSB3 influence in elevation of goblet cell number to prevent damaging effect of exhaustive running on gut morphology. Increase in goblet cells and healthy changes in the gut after probiotic supplementation have been reported by other authors (Desantis *et al.* 2019). Thus, the mechanisms responsible for goblet cell proliferation with various probiotic strains warrant further investigation.

Our data showed significant decrease in expression of all assayed TJ proteins in forced running rats, pretreated with PBS (PEx group), in comparison with other groups. Conversely, BSB3 administration prior to treadmill running prevented the downregulation of all assayed TJ proteins. Efficacy of *B. subtilis* probiotic in protection of TJ proteins expression during immunological stress has been reported in chickens (Gadde *et al.* 2017). The TJ is a protein complex, including occludin, claudins, zonula occludens (ZO) and junctional adhesion molecules (JAM), which maintain the integrity of the intestinal epithelial barrier by sealing adjacent epithelial cells. Previously, we showed damaging effect of heat stress on expression of TJ proteins in rats (Ducray *et al.* 2019). The decrease expression of TJ proteins indicates impaired gut barrier function, accompanied by intestinal permeability (Pastorelli *et al.* 2013; Bischoff *et al.* 2014). It was reported that disruption of intestinal epithelial integrity results in elevation of serum LPS (Van Houten *et al.* 2015) and the release of I‐FABP into serum indicating the presence of enterocyte injury (van Wijck *et al.* 2012).

We found significant increase in serum LPS and I‐FABP only in PEx group of rats, which directly coincided with a lower presence of TJ proteins in this group. Our observations are in line with results of other authors, who observed elevated levels of LPS, similar to florid sepsis occures in ultramarathon runners (Bosenberg *et al.* 1988; Walsh *et al.* 2011) and increase in serum I‐FABP level after high‐intensity exercise (Pugh *et al.* 2017; Sheahen *et al.* 2018). However, BSB3 treatment prevented damage of intestinal barrier and release of LPS and I‐FABP into circulation after exercise. Several research efforts have been launched to identify preventative strategies to minimize gastrointestinal function decrements in athletes (van Wijck *et al.* 2012). Probiotics have been proposed as one of the promising approaches to maintain gut health during exercise and physical activity (Pyne *et al.* 2015), but only few data show efficacy of probiotics in prevention of intestinal barrier integrity. For instance, Lamprecht *et al. *(2012) reported, that 14 days of multi‐strain probiotic supplementation by athletes increased ZO‐1 expression after exercise. However, these authors did not assess other TJ proteins, and alterations in the gut microbiota were not studied. Our finding that short‐term of *B. subtilis* BSB3 consumption significantly protected gut integrity by preventing the loss of TJ proteins following excessive exercise is novel, and we posit that this effect was modulated through positive alterations in the gut microbiota.

Gut microbiota play a significant role in maintaining gastrointestinal barrier integrity (Bischoff *et al.* 2014). Various diseases and disorders are accompained with dysfunctional intestinal barrier and gut dysbiosis (Konig *et al.* 2016; Wang *et al.* 2017). Culture‐based analysis of the gut microbiota in our study revealed significant elevation of haemolytic bacteria, yeasts and *Bacteroides* sp. only in rats from PEx group. We have previously reported that haemolytic bacteria are elevated in rats after exposure to heat stress (Ducray *et al.* 2019). Additionally, others have reported that elevations in haemolytic bacteria and yeasts signify the occurrence of dysbiosis in weanling pigs (Poulsen *et al.* 2017), patients with inflammatory bowel disease (Sokol *et al.* 2017) and individuals with autism spectrum disorders (Iovene *et al.* 2017). *Bacteroides* sp. is a component of the normal gut microbiota, but overrepresentation of these bacteria is associated with different pathological conditions, such as colorectal cancer (Zou *et al.* 2018), bacteraemia (Lassmann *et al.* 2007) and septic arthritis (Wexler 2007). We also found significant increase in *Lactobacillus* sp. and *Bifidobacterium* sp. in rats from the PEx group compare to other groups. These data are in accordance with results of Queipo‐Ortuno et al. (Queipo‐Ortuno *et al.* 2013) about elevation the number of *Lactobacillus* sp. and *Bifidobacterium* sp. in rats after exercise, although other authors reported decrease in relative abundance of *Lactobacillus* bacteria after strenuous physical activity (Chaves *et al.* 2018). Increase in *Lactobacillus* sp. in rats after heat stress was demonstrated in our previous work (Ducray *et al.* 2019). We can speculate that increase in *Lactobacillus* and *Bifidobacterium* in PEx rats is a response of the gut microbiota to a significant elevation of conditionally pathogenic bacteria, as an attempt to normalize microbial balance.

Results of pyrosequencing showed that *Firmicutes*, *Bacteroidetes* and *Actinobacteria* are the dominant phyla in all groups of rats, which correspond to observations by other authors (Byerley *et al.* 2017). No difference in phyla presentation was found in different groups, but the *Firmicutes* to *Bacteroidetes* ratio was highest in the BEx group. Analysis of the gut microbiota on the genus level revealed a substantial increase in *Firmicutes* (*Allobaculum, Olsenella* and *Faecalibacterium*) in rats from the BEx group in comparison with sedentary PCont rats. These bacteria are known to be effective producers of short‐chain fatty acids (SCFA) (Shing *et al.* 2014; Kubasova *et al.* 2018), particularly butyrate, which is the primary energy source for colonocytes, and can thus protect intestinal barrier (Donohoe *et al.* 2011). *Allobaculum*, *Olsenella* and *Faecalibacterium* have been associated with a reduction in gut inflammation and promotion of intestinal health (Wong *et al.* 2013; Rosa *et al.* 2018). In clinical trials intake of probiotic by runners significantly increased the abundance of *Faecalibacterium* and resulted in relief of stress‐associated symptoms (Sawada *et al.* 2019). Treatment with a probiotic, containing *Lactobacillus acidophilus*, *Bifidobacterium longum* and *Enterococcus faecalis* resulted in the improvement of the gut microbiota in obese mice with substantial elevation of *Allobaculum* and *Olsenella* (Kong *et al.* 2019). Data, obtained in our study, demonstrate that all tested parameters of gut health in rats from BEx group were similar to sedentary controls, and these parameters were generally more improved in BEx *vs* PEx rats. We also observed a substantial decrease in *Flavonifractor* bacteria in BEx rats. Positive associations have been reported between *Flavonifractor* and inflammatory markers in patients with pemphigus vulgaris disease (Huang *et al.* 2019). An increased abundance of *Flavonifractor* has also been reported in patients with systemic lupus erythematosus and bipolar disorder (He *et al.* 2016; Coello *et al.* 2019). *Flavonifractor* has been identified as pathogen for cholecystitis and a causative for infections in immunosuppressed patients (Berger *et al.* 2018). Thus, the effects BSB3 had in preventing increases in intestinal *Flavonifractor* levels following a strenuous exercise may promote widespread health benefits in individuals who chronically engage in high levels of physical activity.

Unlike beneficial changes in microbiota in BEx animals, pathogenic bacteria were dominant in the microbiota of PEx rats. In this regard, a significant elevation of *Enterorhabdus*, and appearance of *Facklamia* and *Veillonella* was found in this group of rats. *Enterorhabdus* has been implicated in the development of obesity (Wang *et al.* 2016), autism spectrum disorders (de Theije *et al.* 2014), and is predominant in prediabetic patients (Yang *et al.* 2015). *Facklamia*, alpha‐haemolytic bacteria, have been shown to cause bacteraemia (Rahmati *et al.* 2017), acute cystitis and sepsis (Mostafa *et al.* 2019). *Veillonella* species have been reported to be aetiological factor of endocarditis (Saladi *et al.* 2017) and associated with Crohn’s disease (Kugathasan *et al.* 2017). Significant increases in these bacteria have been observed in the gut microbiota of children with inflammatory bowel syndrome (Rigsbee *et al.* 2012) and in patients with primary sclerosing cholangitis (Kummen *et al.* 2017). Obtained data about predominance of pathogenic bacteria in the gut microbiota of PEx rats agree with the results of our culture‐based analysis and with our previous finding, which indicated significant elevation of pathogens in the gut microbiota of rats after heat stress (Ducray *et al.* 2019). Our results showed considerable decrease in *C. arthromitus* in rats after running (groups PEx and BEx) in comparison with sedentary group PCont. *Candidatus arthromitus* is associated with healthy gut, maturation of the immune system, and protection against pathogens (Hooda *et al.* 2016; Hedblom *et al.* 2018). Reduction in *C. arthromitus* in treadmill‐exercised rats indicates specific depressive effect of endurance running on this bacterium.

Analysis of the gut microbiota in BCont rats revealed significant elevation of *Olsenella* bacteria, the same as in BEx group. We conclude that increase in these bacteria in rats, pretreated with BSB3, is a result of beneficial stimulation by this strain. Additionally, abundance of *Parasporobacterium* in BCont group decreased. This is a positive change in the gut microbiota, given that the predominance of *Parasporobacterium* is associated with Down syndrome (Biagi *et al.* 2014) and with inflammatory bowel syndrome (Shankar *et al.* 2015).

This study demonstrated that forced treadmill running results in significant pathological changes in morphology and function of the gut, as well as in the gut microbiota. Our results for the first time showed that short‐term pretreatment of animals with *B. subtilis* BSB3 beneficially modulates the gut microbiota and as a result, prevents adverse effects of excessive exercise. Indeed, our data are in rodents following an acute bout of unaccustomed treadmill exercise. We believe that BSB3 has a great potential for the development of a valid approach for prevention of excessive exercise‐induced adverse effects.

## Conflict of Interest

No conflict of interest declared.
